# Comparison of the learning curve of intraoral scanning with two different intraoral scanners based on scanning time

**DOI:** 10.1186/s12903-023-02963-7

**Published:** 2023-05-09

**Authors:** Ivett Róth, Péter Hermann, Viktória Vitai, Gellért Levente Joós-Kovács, Zoltán Géczi, Judit Borbély

**Affiliations:** Department of Prosthodontics, Semmelweis University, Szentkiralyi street 47, Budapest, 1088 Hungary

**Keywords:** Intraoral scanner, Learning curve, Scanning time

## Abstract

**Background:**

The appearance of intraoral scanners (IOSs) in dental offices was an important milestones for the digital innovations in dentistry. Knowing the learning curve for intraoral scanning is crucial, because it can serve as a guideline for clinicians before buying a new IOS. The aim of the present in vivo study was to determine the learning curve required by dental students for intraoral scanning with the 3Shape Trios 4 IOS and the CEREC Primescan IOS, based on scanning time.

**Methods:**

A total of 20 dental students with no previous experience in intraoral scanning participated in the present study. 10 students scanned with Trios 4® IOS (TRI) and 10 students took digital impressions with Primescan® IOS (CER). Every student created 15 digital impressions from patients. Prior to taking the impressions, theoretical and practical education was provided. The total scanning time included the upper and lower arches as well as bite registration, for which average values were calculated. Statistical analysis was performed using the Stata package with a mixed-effects generalized least squares regression models.

**Results:**

The average total scanning times were the following: TRI – 205 s for the 1st impression, 133.6 s for the 15th, CER – 289.8 s for the 1st impression, 147 s for the 15th. The model-based estimate of the difference between the two in case of TRI was 57.5 s, and in CER was 144.2 s which is a highly significant improvement in both cases (*P* < 0.0001). The slope of the scanning time vs. learning phase curve gradually approached flatness, and maintained a plateau: TRI – from the 11th measurement and CER – from the 14th measurement onward.

**Conclusions:**

Given the limitations of the present study, we found difference between the learning curve of scanner types which are operate various principle of imaging. In case of the TRI fewer digital impressions (11 repeating) were sufficient to reach the average scanning time of an experienced user than using CER (14 repeating).

**Trial registration:**

The permission for this study was given by the University Ethics Committee of Semmelweis University (SE RKEB number: 184/2022).

## Background

New trends in dental treatment have appeared with the progression of digital innovations in dentistry. New materials with improved physical and esthetic properties have appeared in the dental market which were previously unable to be prepared by conventional technologies [1–3]. The appearance of such new materials was marked by the arrival of new devices, such as laboratory scanners and computer numerical control (CNC) milling machines. Digital innovations in dentistry started in dental laboratories, from which in of the dental computer-aided design/computer-aided manufacturing (CAD/CAM) workflows [1, 4]. One of the most important milestones for the digital innovations in dentistry was the appearance of intraoral scanners (IOSs) in dental offices, with which dentists were able to participate in the digital world.

The first laboratory-connected IOS was introduced in 2007 by the Massachusetts Institute of Technology [5]. Since then, numerous IOSs have appeared in the dental market, leading many scientific publications to investigate the different properties of IOSs compared to traditional impression taking [5–7]. Most of the studies have focused on the acceptance of digital IOSs by members of the dental team as well as their patients [8–15], the accuracy of IOS devices [14, 16–22], and the time required for digital impressions to be made [8, 10, 14, 23–28]. Recent studies have shown that dental students, dental hygienists, and dentists prefer digital impressions over traditional impression materials [11–14]. Making an obvious conclusion regarding accuracy based on the available literature can be difficult. The precision of indirect restoration is not acceptable > 200 μm, as the clinically acceptable range for the marginal fit of fixed restorations is 50–120 μm [29]. Based on previous studies, the acceptable range was achievable with IOSs using short-span bridges (max 1–4-unit restorations) [10, 24, 30–33]; however for long-span restorations, the conventional impression-taking method might be a better option [7, 18, 23, 34]. Recent publications have stated that digital impressions made using updated software and newer generations of IOS devices could be as accurate as conventional impressions, and that full mouth rehabilitation may be possible based on these virtual models [35–39]. Manufacturers often create new versions of their software to improve the properties of their devices annually, or even more frequently [40]. The software updating means that the IOS received an updated version of the software, although the hardware of the device remained the same [41–43]. A new generation, however, meant that the manufacturer had created a brand-new IOS that worked with new software [7].

The scanning time of the impression taking process was one of the most examined parameters of IOSs, and studies verified that the time it took to create impressions with digital IOSs was less than that of the traditional method [10, 23–25, 27, 30, 44]. In most publications, digital impressions were made by an “experienced dentist” [45–48], although it was not clear how many scans were performed by the dentist before they started performing exams on patients. Knowing the learning curve for intraoral scanning is crucial, because it can serve as a guideline for clinicians before buying a new IOS device. The other important aspect of determining the learning curve is the scientific factor: it can be helpful if “experienced user” is defined objectively in publications. The learning curve for intraoral scanning has been evaluated in a few previous studies [49–53], and our workgroup has previously examined the learning curve based on scanning time and image number [49].

Based on the available literature, it was shown that the learning curve for intraoral scanning could be described sufficiently based on the scanning time of the digital impression taking process [49–52]. The flat phase of the learning curve is the section in which additional improvement in the examined parameter cannot be detected [54]. In our previous study, the flat phase based on the average image number, and scanning time was never reached, because 10 digital impressions were not sufficient for the students to be comparable with an experienced user [49]. Based on these results, further measurements were necessary to determine the flat phase (the “experienced user” term) for long-term clinical application and for scientific reasons. The results of a previous study indicated that flat phase is located somewhere between the 10th and 15th digital impression [53]. Furthermore, based on literature there is less information about the learning curve of chairside systems [50]. The CEREC system was the first chairside system in the dental market and many generations of the device were conducted from their first appearance in 1985 [55–57]. The Primescan is the newest generation of CEREC IOs, it was released in 2019 [49, 58].

The aim of the present in vivo study was to determine the learning curve required by dental students for intraoral scanning with the Trios 4 intraoral scanner (IOS) and the Primescan IOS, based on scanning time. The learning curve for dental students was ascertained from the total scanning time during 15 full-arch digital impressions with both IOSs. The null hypothesis was that the flat phase of the learning curve could not be reached when taking 15 digital impressions using Trios 4 or Primescan IOSs.

## Methods

The present study involved 20 dental students. The students were between four and eight semesters into their graduate dental studies, with no experience in intraoral scanning. Each student made 15 digital study impressions (a total of 300 impressions), which included full-arch scans of the upper and lower jaws with bite registration in the intercuspidal position. The students took the digital impressions from other dental students. The permission for this study was given by the University Ethics Committee of Semmelweis University (SE RKEB number: 184/2022). The inclusion criteria were as follows: no missing teeth (full dentition except for wisdom teeth); no orthodontic brackets or fixed restorations; intact soft and hard tissue (no caries/gingivitis/periodontitis); good oral hygiene; and normoclusion. During the impression taking, dental assistants helped the students, who were supervised by dentists with more than three years of experience in digital impression taking. 10 dental students took digital impressions using a 3Shape Trios 4 IOS® (TRI) (software version: Shape Unite 21.2) and the other 10 students scanned with the CEREC Primescan IOS® (CER) (software version: CEREC software v.5.2.3.) (150 impressions with both IOSs). The division of the students into the two comparative groups was blinded randomized. The Trios 4 IOS was a pod system, meaning that the device was connected via Universal Series Bus (USB) to a high-performance laptop [5, 59]. The Primescan was a cart version device with built-in computer [5, 60]. Before taking the impressions, the IOS devices were calibrated following the manufacturer’s instructions using calibration tools (TRI: calibration tip, CER: calibration neck) were used [59, 60]. The virtual models were created according to the manufacturer’s instructions. A retractor (Optragate, Ivoclar) was used to provide better visualization during the impression taking. The patients were in a supine position for the upper and lower jaw scans, and in a sitting position for bite registration.

Theoretical and practical education in the same way of both devices was provided before the start of the impression taking process. During the theoretical education, the operation of the IOSs were presented step-by-step, and the scanning strategies were demonstrated. Knowledge of the scanning paths is crucial, because the accuracy of virtual models and the working time of intraoral scanning are both dependent on the scanning strategy [47, 61, 62]. In the case of the Trios 4 IOS, the suggested scanning path is as follows: the upper and lower jaws both start at the occlusal surface; when scanning maxilla, move from the occlusal, to the buccal, to the palatal surface; and when scanning the mandible, move from the occlusal, to the lingual, to the buccal surface [59, 63]. In case of the Primescan IOS, the recommended scanning strategy is the following: the first surface is the occlusal, then continue with the buccal and palatal/lingual, finally the impression-taking is ended with the scanning of approximal areas [60]. For practical training, the dental students used the IOSs to practice digital impression taking, only after they had received the theoretical training. Each student took digital impressions from a polymethyl methacrylate model in an articulator (upper and lower jaw scan with bite registration). Subsequently, in vivo scanning was demonstrated to the students by an experienced dentist (one of the supervisors), and as the last part of practical education, each dental student made an in vivo digital study impression.

**Fig. 1:**
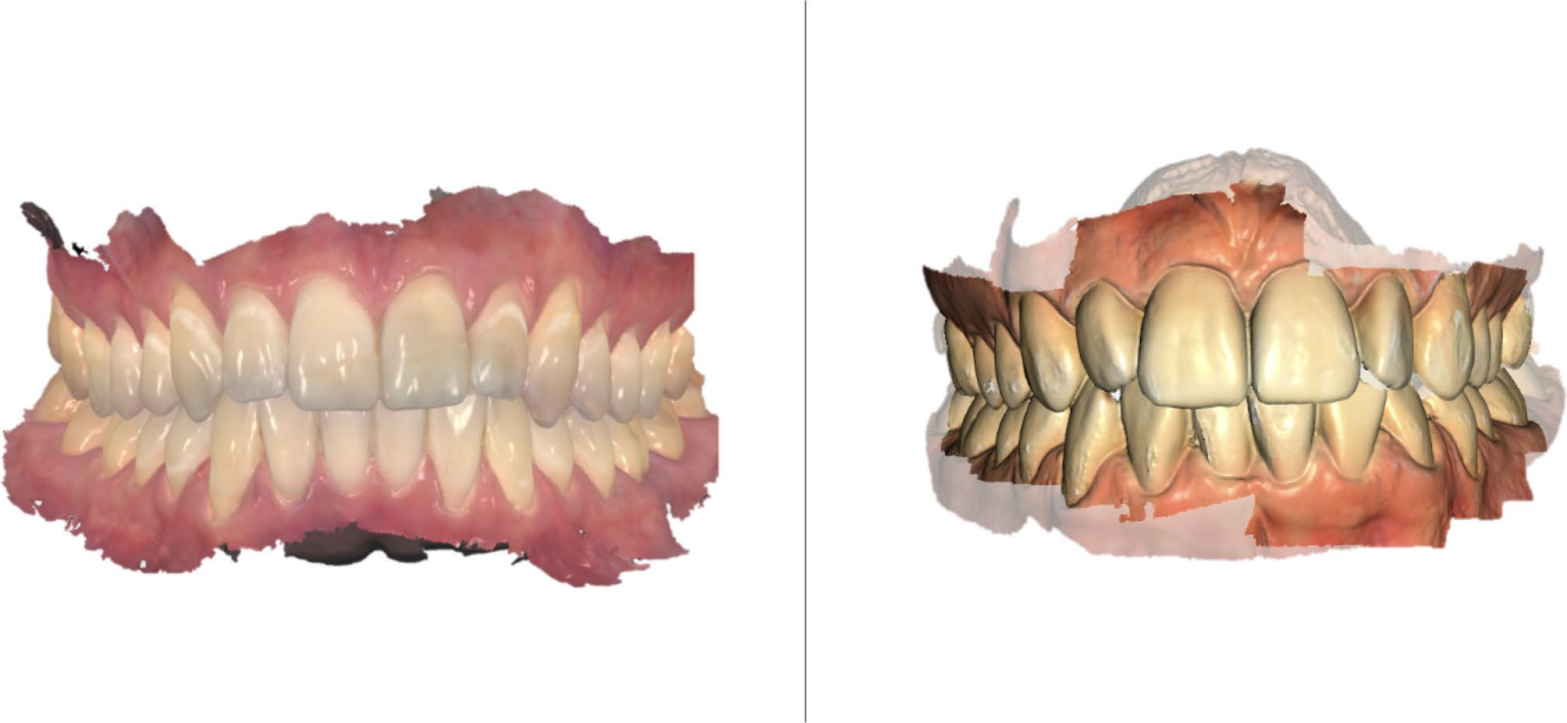
Virtual models created by Trios 4 IOS (left side) and Primescan IOS (right side)

During the measurement portion of the study, the digital impressions were obtained consecutively by each dental student, and data were collected between October and November 2022. Each digital impression was considered acceptable if every surface of every tooth was scanned and bite registration was successfully performed. (Fig. 1)

**Fig. 2:**
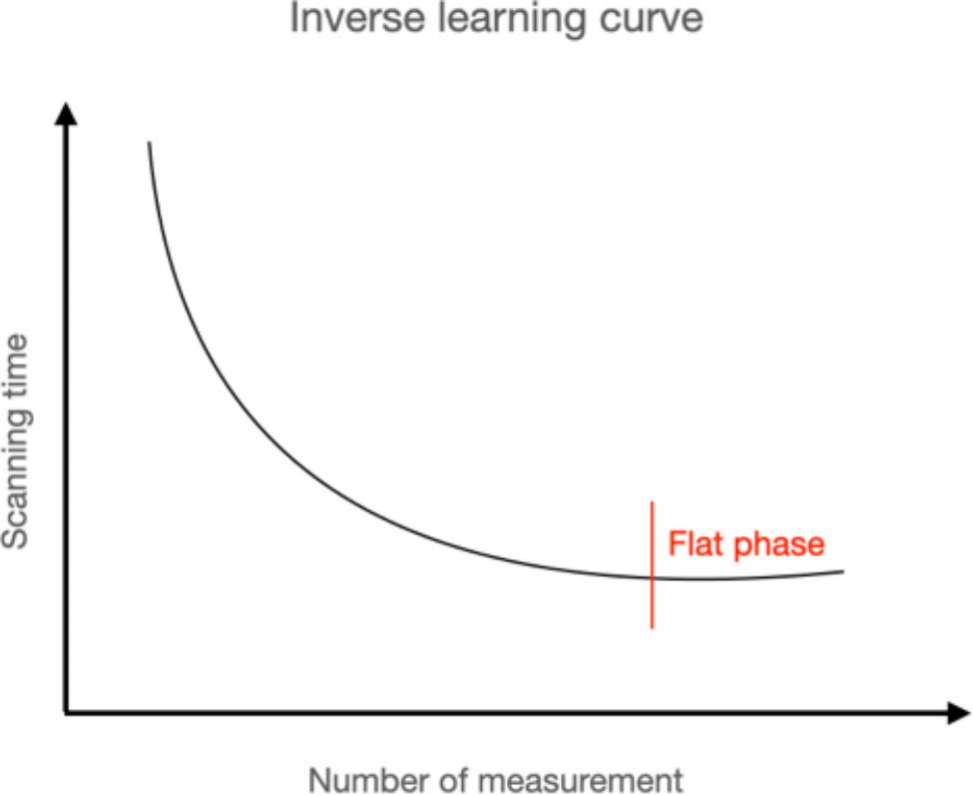
Inverse learning curve: the scanning time is presented on the x-axis, and the number of measurements is described on the y-axis. The flat phase is the section of the learning curve in which additional improvement in the examined parameter cannot be detected

Additional images were added in the case of missing surfaces (e.g., approximal surfaces of the teeth). The total scanning time for the upper and lower arches and bite registration were measured with a stopwatch, and the average values were calculated. After statistical evaluation of the results, the learning curves for the dental students in terms of scanning time were determined. A learning curve is a visual representation of the rate of education through repeated experiences. Various types of learning curves have been described in the available literature; however, the most commonly used is the classic type. The classical learning curve can be divided into three sections: positive, middle, and negative learning periods. The curve starts from learning level zero, and during the positive growth period, the learning speed increases constantly, while in the middle section, the pace of learning is uniform. During the negative period, the rate of learning decreases and the learning curve ends in a flat phase. The flat phase of the learning curve is the section where additional improvement of the examined parameter cannot be detected. In the present study, we observed an inverse learning curve of intraoral scanning based on scanning time. The number of scans was presented on the x-axis and the time required was demonstrated on the y-axis [54]. (Fig. 2) Statistical analysis was performed with the Stata software package using a mixed-effects generalized least squares regression model of natural log-transformed total scanning time as the outcome against the sequential number of measurements, a continuous explanatory variable analogous to the learning stage. A student identifier was used for the random effects. The relationship curve was created by adding a squared term for the measurement number if its effect was significant, α = 0.05. Hausman’s specification test was used to confirm that fitting a random effects model was justified.

## Results

**Fig. 3:**
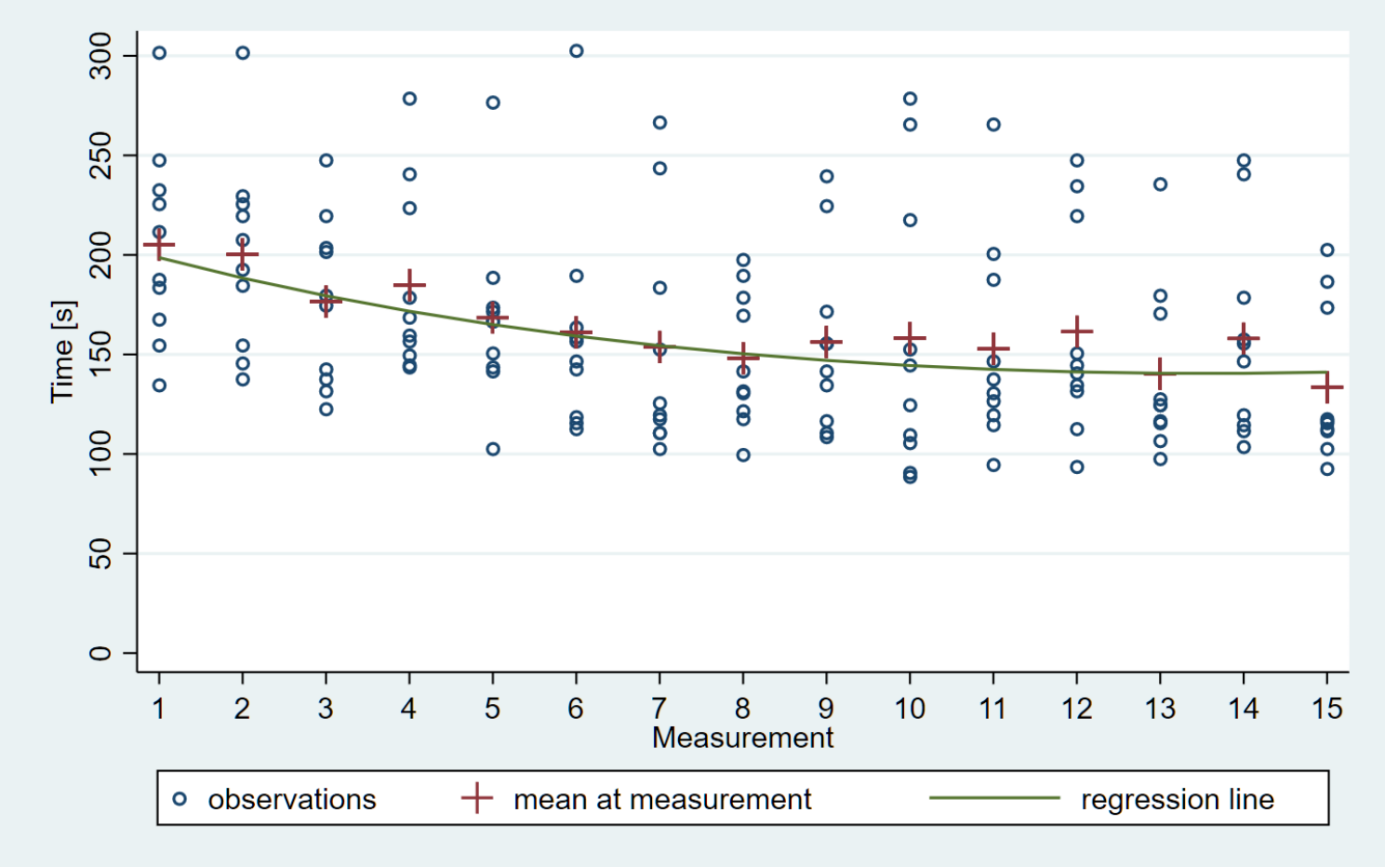
Inverse learning curve of intraoral scanning with Trios 4 IOS based on total scanning time; the model-based estimate of the difference between the 1st and 15th measurements was 57.5 s (95% confidence interval [CI]: 76.0–39.1), a very significant improvement (P < 0.0001)

**Fig. 4:**
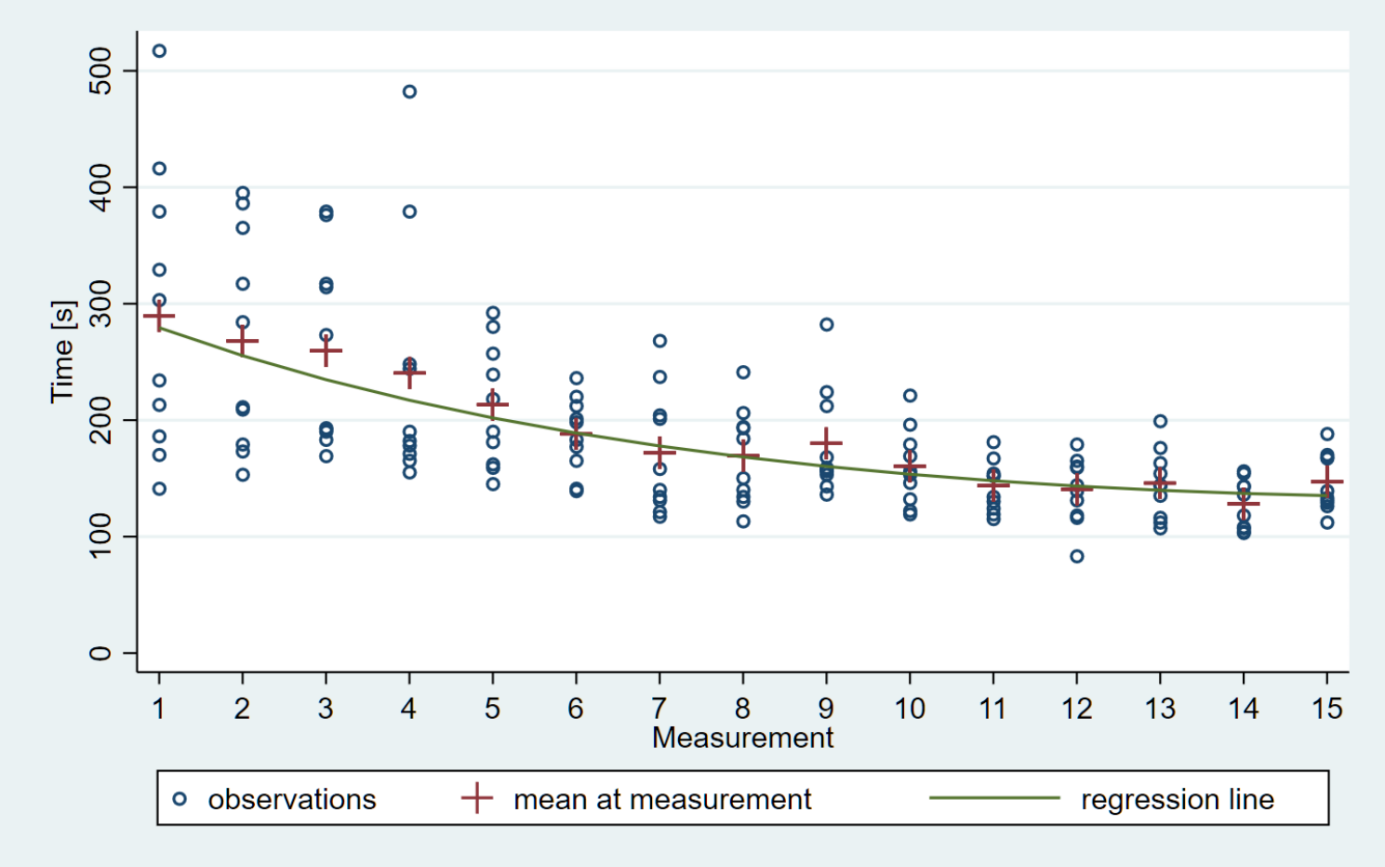
Inverse learning curve of intraoral scanning with Primescan IOS based on total scanning time; the model-based estimate of the difference between the 1st and 15th measurements was 144.2 s (95% confidence interval [CI]: 76.0–39.1), a very significant improvement (P < 0.0001)

From the 150 impressions, the average total scanning times were the following: TRI – 205 s for the 1st impression, 133.6 s for the 15th, CER – 289.8 s for the 1st impression, 147 s for the 15th. The model-based estimate of the difference between the two in case of TRI was 57.5 s, and in CER was 144.2 s (95% confidence interval [CI]: 76.0–39.1) which is a highly significant improvement in both cases (*P* < 0.0001). (Figures 3 and 4)

**Fig. 5:**
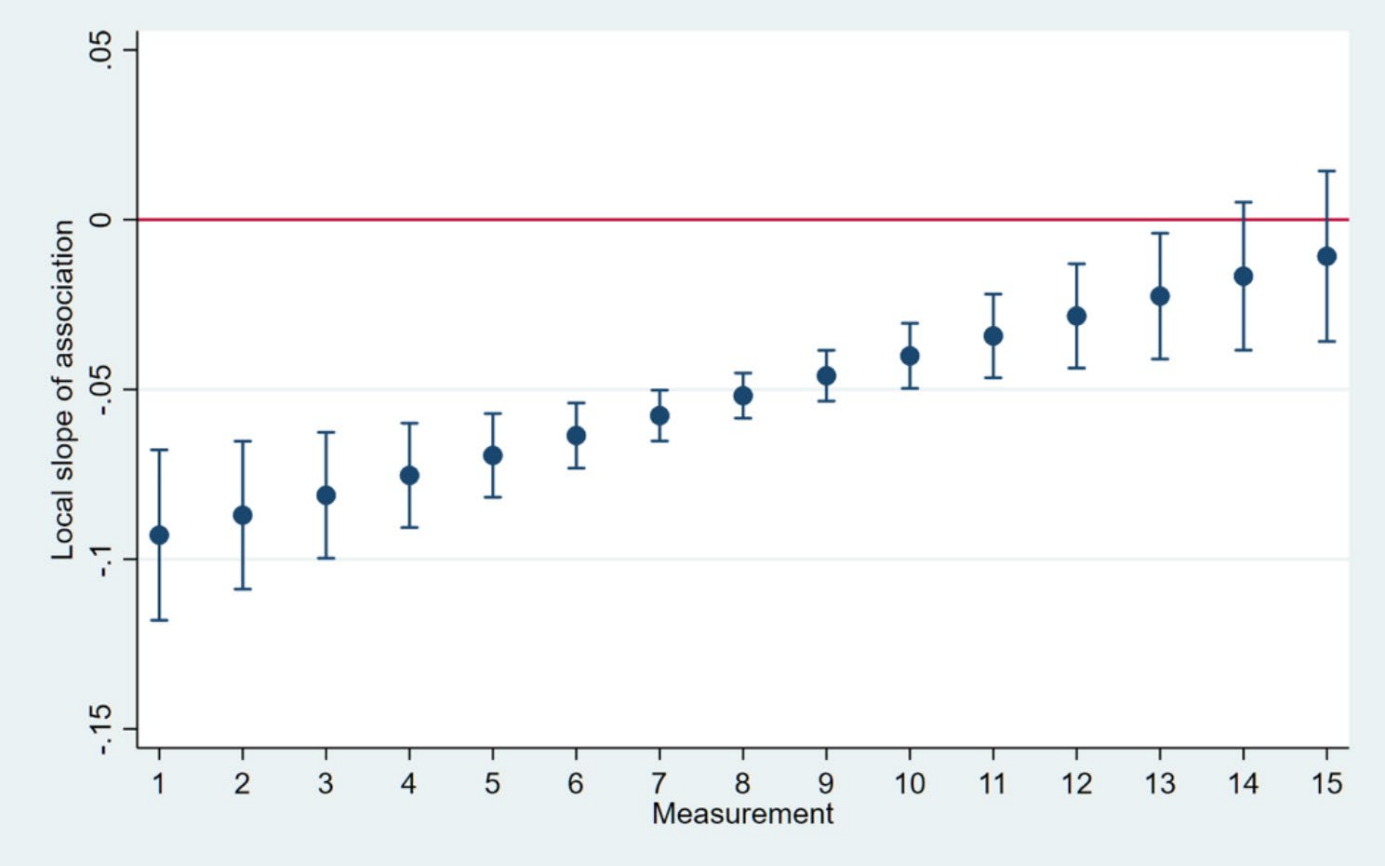
The slope of the scanning time vs. learning phase curve was significantly negative throughout the first 10 impressions, gradually approaching flatness and maintaining a plateau from the 11th measurement onward in case of Trios 4 IOS

In case of learning curve of Trios 4 IOS the slope of the scanning time vs. learning phase curve was significantly negative throughout the first 10 impressions, gradually approaching flatness and maintaining a plateau from the 11^th^ impression onward. (Fig. 5)

**Fig. 6:**
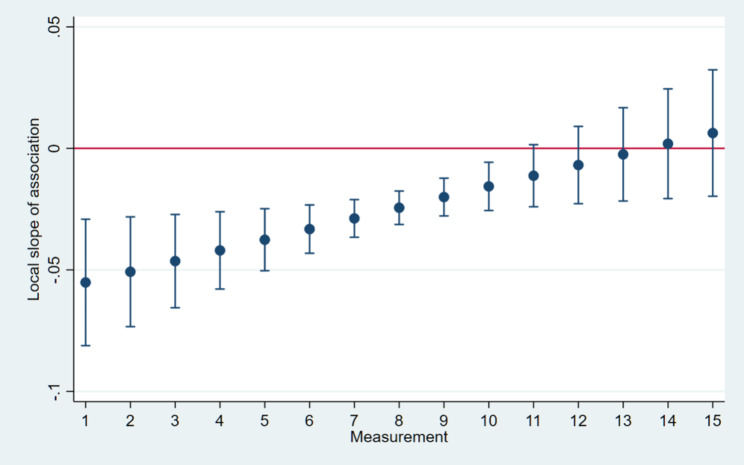
The slope of the scanning time vs. learning phase curve was significantly negative throughout the first 13 impressions, gradually approaching flatness and maintaining a plateau from the 14th measurement onward in case of Primescan IOS

In case of Primescan the slope was significantly negative throughout 13 impressions then approaching flatness (plateau) from the 14^th^ impression taking. (Fig. 6)

The null hypothesis stated that the flat phase of the learning curve could not be reached when taking 15 digital impressions using Trios 4 or Primescan IOSs; however, based on our results, the null hypothesis was rejected from the point of view of both scanner devices, as the learning curve did, in fact, reach a flat phase (plateau).

## Discussion

Every aspect of dentistry, along with the steps of various dental workflows, have been radically changed by the introduction of dental CAD/CAM technology. The implementation of IOSs into the digital workflow has transformed the treatment processes relating to prosthodontics [5, 7, 64]. Digital impression taking has many advantages: less working time, decreased gag reflex, sustainability (elimination of silicon and gypsum dental materials, less waste during the prosthetic workflow), and better communication between patients and dental technicians [5, 64–66]. The workflow of traditional model making often results in inaccuracies of restorations, which result from multistep gypsum pouring errors [67]. This prosthetic workflow failure can be eliminated by utilizing a digital impression recording technique. Furthermore, impression mistakes would be noticed during the impression-taking process (in contrast with the traditional method, when most of the errors are detected after cast making); therefore, the impression can be corrected immediately [5]. Digital impression talking also has disadvantages, such as coming with a variety of costs (software updates/closed systems fees, and the costs of IOSs) [68, 69]. Furthermore, detecting deep margins or difficult bite registrations could be challenging with IOSs [5, 70, 71]. The accuracy of the digital impression could be influenced by many factors such as type, principle and ergonomic design of the IOS device [72–76], patient-related parameters (saliva flow, ability of mouth opening, abnormalities in the dentition) [5, 7, 23] and the dimension of the digital impression (quadrant or full arch scan) [77], the ambient light [78] and the efficiency of the operator [23, 79]. Based on literature, the accuracy of implant and traditional digital impressions could be different [5, 36, 64, 80] and presumably the learning curve of them also differ [81].

IOSs have a learning curve similar to traditional impression taking methods, and a certain degree of experience is required to operate an IOS. The learning curve for intraoral scanning is an unexplored area of dental research, and the first scientific studies on this subject were published only a few years ago [49–53]. In 2020, our workgroup published an article on the learning curve of intraoral scanning procedures. The aim of our previous study was to determine the flat phase of the learning curve of intraoral scanning over the course of 10 digital impressions [49]. The flat phase of the learning curve is the section where additional improvement of the examined parameter cannot be detected [82]. In our previous study, 10 dental students each made 10 digital impressions after a training session, and the scanning time and number of images for each digital impression were measured. Based on the average scanning time and image number the learning curve of the students’ scanning process could be established. In that study, we found that the average scanning time tended to decrease between the 1st and 10th digital impressions, with a decrease of approximately 8 min, which was quite a significant difference. The number of images is the number of images created by the IOS during scanning, and in the case of the Trios ISO devices (Trios 3, 4 and 5), a number of images appeared on the computer screen after the scanning process [49]. The average number of images is difficult to assess in digital dentistry, because there are very few relevant studies in the literature. Research has proved that more precise digital impressions were obtained using an IOS with a larger scanner head, as a larger area could be scanned at the same time; therefore, there were fewer merged images, and the virtual impression had higher precision and trueness values [72, 73]. In our previous study, there was not an obvious downward trend in the number of images per impression between the 1st and 10th digital impression. From the 1st through the 6th impression, a decreasing tendency was observed; however, after that point, the number of images increased. As dental students became more confident in their digital impression taking, they worked faster; however, they started to randomly miss important areas, such as the approximal surfaces. Due to the missing regions, the students had to perform additional scanning to fill the gaps. The extra scanning was not evident in the scanning time results because of the students’ significant acceleration in speed, but it could be seen in the total number of images per impression [49]. In our previous, it was proved that the learning curve of intraoral scanning could be described based on average scanning time, although the flat phase was not reached in that study, because 10 digital impressions were not sufficient to reach the average scanning time of an experienced user. In the present study, the number of measurements (number of digital impressions) was increased to determine the flat phase of the intraoral scanning learning curve. Furthermore, beside Trios IOS, an additional IOS device was involved to our study: a chairside system, CEREC Primescan. Each dental student obtained 15 digital impressions, and the scanning time of the digital impressions was measured. From these measurements, we found that the flat phase was reached at the 11th impression in case of Trios and at the 14th impression using Primescan, which was sufficient to reach the level of experienced users in terms of scanning time. Based on the results of our present study, in case of Trios IOS only one additional digital impression-taking needed to reach the plateau phase of the learning curve, although presumably it was caused by the software and hardware updates of the IOSs. Based on literature the software updates and appearance of new generations of IOSs in the dental market can improve the properties of the devices, due to that the learning curve of intraoral scanning can be shortened [41, 42]. In the present study, the scanning times of the upper and lower jaws and bite registration were measured and recorded, while the computer time was not included. The computer time refers to the software utilization time, when the operator fills out the digital worksheet (chooses the type of impression) and changes between the upper and lower impressions. The time required for scanning is not affected by computer time, because the computer time does not change significantly after a training session [52].

Optical impressions in the present study were made using two different IOSs: a Trios 4 Pod IOS which operates on the principle of confocal laser scanning technology and a Primescan which uses triangulation to create the virtual models [7]. In the case of Trios scanners (Trios 3, 4, and 5), the IOSs create a digital model of a special subtype of confocal technology: ultrafast optical sectioning. During scanning, an ultrafast optical sectioning IOS applies an illumination pattern and light oscillation to the object for faster imaging [5, 64, 83]. The principle of confocal laser scanning microscopy is user sensitive; therefore, the proficiency of the operator during intraoral scanning is important. If the operator does not follow the recommended scanning path (scanning strategy), and makes sudden movements during the digital impression taking, artifacts will be created, which can cause inaccuracies in the digital model. The IOS handpiece needs to be guided slowly, without any thrill, and changing the distance between the scanner head and the tooth surface as little as possible [64]. Another limitation of laser scanning microscopy is the wide optics; therefore, the IOS requires a larger and wider scanner head for sufficient mapping of the surface, which is uncomfortable for the patient and can be inconvenience during clinical use [67]. The principle of optical triangulation uses light strips to illuminate the surface of the teeth and light reflexion is recorded by a complex camera system [5, 74]. Based on literature, there were differences between the accuracy of IOS devices which works by the principle of confocal laser scanning and triangulation [74]. In our study, we have only used two IOSs: the CEREC system was the first chairside system in the dental market and the Trios scanners are one of the most popular lab-connected intraoral scanning systems [5, 56, 57]. Furthermore, these devices are working on different mapping principles therefore they are appropriate to compare the learning curve of different working principles of IOSs [5]. The learning curve for intraoral scanning may be different depending on the IOS used, but it not only depends on the principles of the imaging technique, but also on the type of scanner. This statement is supported by a study conducted by Kim et al. in 2016, who found that although the imaging principles of Trios and iTero IOSs is the same, their learning curves were different [51]. Zaraus et al. published a study in 2021, in which the time requirement for intraoral scanning was determined in different age groups (dental students ≤ 25 years, dentists ≥ 40 years, and a control group of experienced IOS operators with no age limitation) [52]. The participants in their study used the IOS (Trios 3) five times, as follows: introduction of the IOS and baseline scanning, training (3 × 20 min), and a final scanning. The average scanning time of the baseline and final scanning in the group of dental students aged ≤ 25 years was comparable to our outcomes with Trios 4 IOS (3 min 28 s for baseline and 2 min 43 s for final scanning) [52]. Khaled and Al-Hamad investigated the learning curve for intraoral scanning by prosthodontics residents in vitro (nine digital impressions from an upper and lower model with bite registration), and published their results in 2020. The average scanning time of the 9th measurement was 4 min 37 s, meaning that their results were higher than ours with Primescan (2 min 27 s for the 15th impression). The differences probably came from the different generation of IOSs (they used the CEREC Omnicam IOS which is a previous generation of the device ) [50]. In 2016, Kim et al. conducted scientific research involving 29 dental hygienists who each made 10 digital impressions. The average impression-taking time using the Trios 3 IOS was 14 min 25 s. Their study was published in 2016; therefore, the software version that they used was older than that used in our study. Furthermore, they used a previous generation of the Trios IOS (Trios 3) [51]. In our present study, the same IOSs (Trios 4 Pod and Primescan) were used for every digital impression, which were the newest hardware version available on the market at the time, operating with the latest software version and the best performance computer recommended by the manufacturers [84]. Furthermore, the suggested scanning path of each IOSs was followed for every impression.

Using the Trios 4 IOS, the plateau phase of the learning curve was reached more quickly: 11 digital impression-taking was enough to reach the average scanning time of experienced users, however, with Primescan IOS the examiner students needed to scan 14 times for that. It can be hypothesized that the reason behind it is the different operating principle of the IOSs which can influence the scanning time and the ergonomic properties of the IOSs. The Primescan is heavier than Trios 4 IOS and the scanning speed of students can be influenced by this factor (harder to handle the handpiece of the device) [28]. The reason of the handpiece’s weight is the operating method of the scanner: the postprocessing starts in the IOS handpiece and the virtual model can be radically clear without trimming, furthermore, there is no limit to the number of digital images that can be acquired [58].

The present study did have some limitations. Digital impressions were taken on dental students, who could tolerate the scanning process better than an average patient in a real clinical setting. Another limitation was that the operators of the IOSs were dental students, which could have influenced the results. Our results can be hardly reproducible in everyday clinical practice because an average clinician does not have the opportunity for the extended education and supervised mentoring process provided to the students who participated in our study. The learning curve for dentists can differ from that for dental students. Furthermore, the individual competency of students could also influence the scanning time of impression taking. In our study, the exclusion and inclusion criteria of the patients were defined, nevertheless, the patient related factors (e.g. abnormalities in the dentition, occlusion forms, ability of mouth opening) could influence the measurements.

## Conclusions

In the present study, the scanning time of both intraoral scanner devices (Trios 4 and Primescan) decreased during 15 digital impressions obtained by dental students. The difference between the first and last average total impression taking time showed a very significant improvement in both cases. The learning curve approached flatness and maintained a plateau with less practice using the Trios 4 IOS than Primescan. In case of the Trios 4 IOS fewer digital impressions (11 repeating) were sufficient to reach the average scanning time of an experienced user than using Primescan IOS (14 repeating). Given the limitations of the present study, it can be stated that there is difference between the learning curve of dissimilar IOS types which are operate various principle of imaging (TRI: confocal laser scanning microscopy, CER: triangulation).

## Data Availability

The datasets used and/or analyzed during the current study are available from the corresponding author on reasonable request.

## Abbreviations

CAD/CAM: Computer Aided Design/Computer Aided Manufacturing
IOS: intraoral scanner
CNC: computer numerical control
CEREC: Chairside Economical Restorations of Esthetic Ceramic
